# Cook County Medical Society

**Published:** 1856-04

**Authors:** 


					﻿Cook County Medical Society.
At the regular meeting of the Society, held on the first
Tuesday evening in April, the following Officers and Delegates
were chosen for the ensuing year:—
Dr. D. LASKIE MILLER, President.
Dr. E. ANDREWS, Vice-President.
Dr. J. II. HOLLISTER, Secretary.
Delegates to the American Medical Association: Drs. J.
Bloodgood, II. Parker, J. H. Hollister, and E. Andrews.
Delegates to the Illinois State Medical Society: Drs. J. W.
Freer, II. A. Johnson, Thos. Bevan, and M. 0. Heydock.
				

